# Randomizing of Oligopeptide Conformations by Nearest Neighbor Interactions between Amino Acid Residues

**DOI:** 10.3390/biom12050684

**Published:** 2022-05-11

**Authors:** Reinhard Schweitzer-Stenner, Bridget Milorey, Harald Schwalbe

**Affiliations:** 1 Department of Chemistry, Drexel University, 3141 Chestnut Street, Philadelphia, PA 19104, USA; bridget.j.milorey@drexel.edu; 2 Center for Biomolecular Magnetic Resonance, Institut für Organische Chemie und Chemische Biologie, Johann Wolfgang Goethe Universität, 60438 Frankfurt, Germany

**Keywords:** Ramachandran distributions, isolated pair hypothesis, nearest neighbor interactions, model peptides, intrinsically disordered proteins

## Abstract

Flory’s random coil model assumes that conformational fluctuations of amino acid residues in unfolded poly(oligo)peptides and proteins are uncorrelated (isolated pair hypothesis, IPH). This implies that conformational energies, entropies and solvation free energies are all additive. Nearly 25 years ago, analyses of coil libraries cast some doubt on this notion, in that they revealed that aromatic, but also β-branched side chains, could change the ^3^J(H^N^H^Cα^) coupling of their neighbors. Since then, multiple bioinformatical, computational and experimental studies have revealed that conformational propensities of amino acids in unfolded peptides and proteins depend on their nearest neighbors. We used recently reported and newly obtained Ramachandran plots of tetra- and pentapeptides with non-terminal homo- and heterosequences of amino acid residues to quantitatively determine nearest neighbor coupling between them with a Ising type model. Results reveal that, depending on the choice of amino acid residue pairs, nearest neighbor interactions either stabilize or destabilize pairs of polyproline II and β-strand conformations. This leads to a redistribution of population between these conformations and a reduction in conformational entropy. Interactions between residues in polyproline II and turn(helix)-forming conformations seem to be cooperative in most cases, but the respective interaction parameters are subject to large statistical errors.

## 1. Introduction

**Figure 1:**
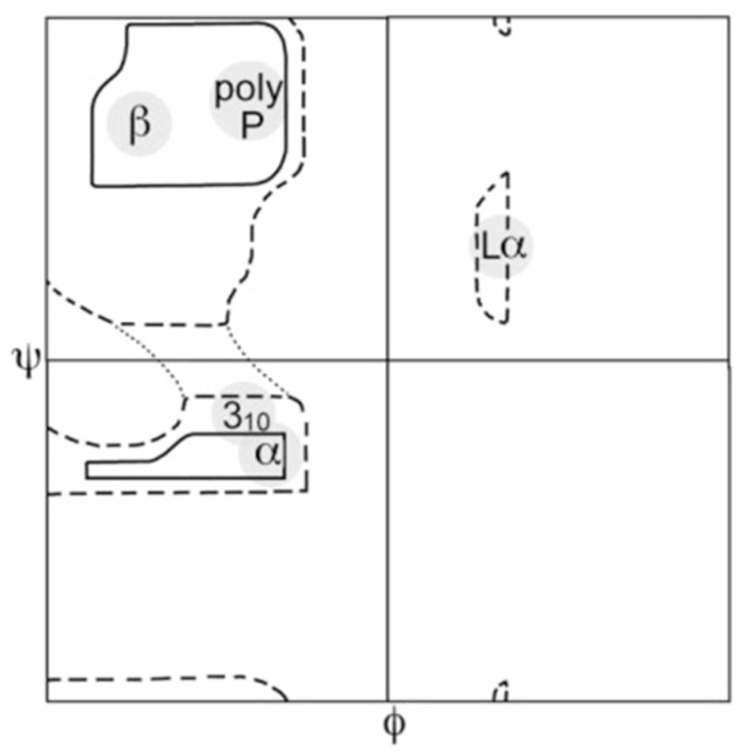
Sterically allowed φ,ψ space proposed by Ramachandran [33]. Solid lines enclose regions allowed by hard-sphere bumps at standard radii; dashed lines show regions allowed with reduced radii; dotted lines add regions allowed if τ (N-C_α_-C’) is relaxed slightly. Ψ and φ values run from −180° to 180°. Taken from ref. [34].

The unfolded state of proteins still attracts substantial interest of protein biophysicists and biochemists. There are several reasons for the increased focus on what for a long period of time was considered a subject of minor interest because of its assumed irrelevance for protein function and the notion that available theories had provided a sufficiently thorough understanding of its basic properties [1,2,3]. One of the motivations for the paradigm change was the discovery of so-called intrinsically disordered proteins (IDPs) which were found to perform important biological functions, particularly in cellular contexts, in spite of the absence of any well-defined secondary structures [4,5,6]. Some of these proteins (and peptides) are prone to self-assembly into amyloid fibrils which are thought to be involved in neurological diseases such as Alzheimer’s, Parkinson’s and Huntington’s disease [7,8]. Alternatively, the self-assembling of IDPs can lead to liquid–liquid demixing and the subsequent formation of intracellular bio-molecular condensates [9,10,11]. In addition to fully disordered proteins, a plethora of semi-disordered proteins have been discovered for which disordered segments play a very important functional role [12,13]. Very much in parallel to the increasing research on disordered proteins and peptides, multiple lines of experimental, computational and bioinformatic evidence have emerged for the notion that the conformational space sampled by individual amino acid residues in unfolded/disordered peptides and proteins do not sample the entire sterically allowed region of the Ramachandran plot (Figure 1) and that the twenty natural amino acid residues exhibit different conformational propensities for the sampling of basins associated with well-known secondary structures [14,15,16,17,18,19,20,21,22]. Generally, work on unblocked GxG type tripeptides (x: guest residue) and related blocked dipeptides has revealed that amino acid distributions differ in terms of their population of polyproline II (pPII) and β-strand conformations, with alanine strongly preferring the former, while valine, isoleucine and, surprisingly, protonated aspartic acid residues were found to prefer the latter [23,24,25,26,27,28,29]. Right-handed helical conformations are generally less populated than assumed for the construction of random coil distributions of polypeptides and proteins. GxG peptides with x-residues exhibiting hydrogen bonding capability were shown to additionally sample conformations that appear in type II’/I β-, inverse γ- and so called asx-turns [24,30]. Overall, the results obtained with short peptides suggest that unfolded and disordered proteins might exhibit much less conformational disorder than generally assumed. A similar picture has emerged from the studies of some coil libraries, but differences between corresponding coil library and peptide distributions are noteworthy [31,32].

If, as Flory assumed, the Gibbs energy landscape of unfolded proteins in an ideal random coil state was defined by the superposition of energy surfaces of their individual residues (isolated pair hypothesis, IPH), the above cited Ramachandran distributions could be used to calculate their conformational distributions in the absence of any non-local interactions [1]. This should indeed be the case in an ideal random coil state where attractive non-local interactions are exactly neutralized by excluded volume effects. Non-local interactions would also be absent if solvent–protein/peptide and repulsive intraprotein interactions favor an extended state in which the surface accessible area of residues is maximized. Interestingly, however, several lines of evidence suggest that even in the absence of non-local attractive interactions, nearest neighbors and potentially even second nearest neighbors are structurally not independent. Early NMR studies suggest that highly branched and aromatic amino acid residues impose a shift towards more β-strand-like conformations of their neighbors [35,36]. However, conclusions from these studies rely in part on changes of only a single NMR-coupling constant (^3^J(H^N^H^α^)) and of chemical shifts for which different causes could be invoked. Jha et al. deduced nearest neighbor interactions (NNIs) from distributions in coil libraries [16]. For alanine, they clearly showed that conformational distributions do not only depend on the nature of neighbors but even more on their adopted conformation and their position (upstream or downstream on the polypeptide chain). For β-branched neighbors of alanine, NNIs are more pronounced if positioned upstream, while aromatic neighbors interact more efficiently if positioned downstream. Their data indicate the presence of cooperative and anti-cooperative interactions between pPII, β-strand and right-handed helical conformations. A very comprehensive coil library-based NNI analysis of a large number of amino acid pairs was carried out by Ting et al. [17]. They removed all residues in regular β-sheets and α-helices from their ensemble. Their Ramachandran plots suggest a massive influence of NNIs on conformational distributions. While alanine, for example, exhibits a high pPII propensity in the presence of downstream proline, substituting the latter with phenylalanine, valine or glutamine substantially increases the right-handed helical conformations at the expense of pPII. The very same effect was observed for various types of upstream neighbors. 

Computational studies of NNIs in short oligopeptides are noteworthy as well. Some results suggest that NNIs involve peptide–solvent interactions. Garcia, for instance, showed that preferred solvation underlies the cooperative interactions between pPII conformations of alanine in oligoalanine peptides [22,37]. Avbelj et al. performed electrostatic calculations to show that the replacement of an alanine by another amino acid residue in oligo-alanine sequences causes changes of the conformational energies of neighbors by solvent-mediated interactions. Pappu and Rose obtained sterically induced NNI for neighbors in helical conformations [38]. Tran et al. used Monte Carlo simulations of blocked host-guest peptides of different lengths. They found that NNIs due to steric repulsion have a tendency to produce more extended structures by favoring conformations in the upper over those in the lower left quadrant of the Ramachandran plot [39]. Zaman et al. observed that the use of different force fields produces rather different NNIs [40]. Since they used an implicit water model, the role of solvation might have been underestimated. 

Thorough experimental investigations of NNIs are rare. Cho and coworkers used UVCD spectroscopy and ^3^J(H^N^H^α^) coupling constant data to investigate NNIs in blocked tripeptides in aqueous solution [41,42]. They interpret spectroscopic data obtained for upstream aromatic neighbors as indicative of shifts towards more extended structures, but the observed changes of ^3^J(H^N^H^α^) coupling constant data could reflect changed propensities as well as changes of basin positions or a combination of both. This issue was explicitly addressed by Toal et al. who used a large set of five J-coupling constants combined with amide I’ profiles in IR, Raman and vibrational circular dichroism spectra to elucidate NNIs in a selected set of unblocked tetrapeptides [43]. They found that the pPII propensity of alanine is indeed reduced by more sterically demanding nearest neighbors, particularly by valine. However, contrary to the observations of Ting et al., the results of Toal et al. indicate a concomitant stabilization of β-strand rather than of right-handed helical conformations. For aspartic acid, NNIs were found to cause the very opposite effect, a stabilization of pPII over β-strand and different types of turn-supporting conformations. For other pairs of amino acid residues (e.g., KL, LK, VK, etc.) they observed that NNIs changed the position of basins rather than their population. More recently, Milorey et al. augmented these studies by investigations of homopeptide sequences in tetra- and pentapeptides (GDDG, GDDDG, GRRG and GRRRG) [44,45]. For R, the presence of like-neighbors produces a stabilization of β-strand conformations, while conformations generally found in i + 2 residues of type I or II’ β-turns are stabilized by D neighbors. 

While the above studies provide valuable information, their results have not yet entered the realm of thermodynamic models and computational studies. Regarding the latter, recent MD simulations for GRRG and GRRRG indicate that currently used force fields and water models do not account for NNI-type cooperativity [44]. As far as theory is concerned, a first attempt of thermodynamic modeling has recently been undertaken by Schweitzer-Stenner and Toal [46]. They found that the above NNIs of alanine and aspartic acid residues with various neighbors are indicative of cooperativity between pPII and β-strand conformations which can actually be read as anti-cooperativity between pPII conformations of adjacent residues. Such effects seem to be even more pronounced at high temperatures, at which one generally observes thermal unfolding of proteins.

In the current study we extend the model of Schweitzer-Stenner and Toal by considering a larger number of nearest neighbor interaction parameters and by adding the peptides investigated by Milorey et al. [44,45] to the data pool of Toal et al. [43]. The latter is augmented further by a conformational analysis of GAFG, GFAG and GKKG peptides which relies on a set of J-coupling constants. The results reveal a complex pattern of NNIs which very much depend on the nature of the interacting residues. Generally, our results confirm the relevance of pPII-β interactions. Furthermore, we found that interactions between pPII and right-handed helical conformations play a role in arginine and aspartic acid residues containing peptides.

## 2. Materials and Methods

### 2.1. Materials

The Ramachandran plots and conformational propensities of most peptides investigated in this study were determined in earlier studies [43,44,45]. Here we augment this data set with experimentally determined J-coupling constants of the cationic tetrapeptides glycylalanylphenylalanylglycine (GAFG), glycylphenylalanylalanyl glycine (GFAG) and glycyllysyllysylglycine (GKKG). Peptides used for ^1^H NMR experiments were custom synthesized by Genscript. Isotopically labeled peptides were synthesized as described by Milorey et al. [44]. The purified peptides were dissolved at a concentration of 5–10 mM in a 90%H_2_O/10%D_2_O solvent. The used D_2_O solution contained 0.1%4,4-dimethyl-4-silapentanesulfonic acid (DSS) which was used as an internal standard. 

### 2.2. NMR Spectroscopy

E.COSY and J-modulated HSQC measurements were acquired to determine the following set of coupling constants: ^3^J(H^N^,C’), ^3^J(H^α^,C’), ^3^J(H^N^,C^β^) and ^1^J(N,C^α^). Details can be found in the paper of Milorey et al. [44] ^3^J(H^N^,H^α^) constants were determined from ^1^H NMR experiments with unlabeled peptides as described by Toal et al. [25].

*Analysis of NMR data*. The Gaussian model used to analyze the experimental J-coupling constant has been described in detail in earlier papers [23,47]. Briefly, we construct Ramachandran plots as superpositions of two-dimensional Gaussian distributions associated with basins that represent different secondary structures. The position, halfwidths and statistical weights of these Gaussian distributions were varied in an optimization procedure for which we calculated conformational averages of J-coupling parameters with published Karplus equations for the five J-coupling constants [48,49,50]. Details of the optimization procedure are given in the Results and Discussion sections. 

### 2.3. Determination of Nearest Neighbor Interaction Energies

A first version of the theory utilized in this study was recently published by our group [46]. In this paper we introduced a one-dimensional Ising model where we considered changes of residue propensities by NNIs between pPII and β-strand conformations induced downstream by the *i^th^* on the (*i + 1)^th^* residue. To this end, we considered conditional probabilities which reflected the probability for the y-residue in GxyG to adopt pPII or β-strand in dependence of whether the x-residue adopted any of these conformations. We did not explicitly consider any NNIs involving helical or turn-like conformations. The transfer matrices used in our model are reminiscent of the helix-coil model of Zimm and Bragg [37]. 

For the current investigation of NNI we present a more complete model which considers interactions between up to four conformations of adjacent residues. In addition to pPII and β-strand, we consider right- and left-handed helical/turn-like conformations. We do not consider changes of the basin positions of pPII and β-strand. Because helix and turn basins are generally weakly populated (with an exception of asx-turns and type I/II’β turn forming conformations in the Ramachandran plot of D residues) [30,45], we combined the mole fraction of conformations with very similar φ but somewhat different ψ-values e.g., right handed helical, type I/II’ β-turn (*i* + 2) forming and inverse γ conformations from the left as well as left-handed helical asx- and γ-turns from the right half of the Ramachandran plot. Hence, we employ 4 × 4 transfer matrices for the calculation of the partition sum of the considered tetra- and pentapeptides. They read as:

(1) $$T_{ji}=\left(\begin{array}{cccc} {\langle pPII|pPII\rangle }_{ji} & {\langle pPII|\beta \rangle }_{ji} & {\langle pPII|t_{l}\rangle }_{ji} & {\langle pPII|t_{r}\rangle }_{ji}\\ {\langle \beta |pPII\rangle }_{ji} & {\langle \beta |\beta \rangle }_{ji} & {\langle \beta |t_{l}\rangle }_{ji} & {\langle \beta |t_{r}\rangle }_{ji}\\ {\langle t_{l}|pPII\rangle }_{ji} & {\langle t_{l}|\beta \rangle }_{ji} & {\langle t_{l}|t_{l}\rangle }_{ji} & {\langle t_{l}|t_{r}\rangle }_{ji}\\ {\langle t_{r}|pPII\rangle }_{ji} & {\langle t_{r}|\beta \rangle }_{ji} & {\langle t_{r}|t_{l}\rangle }_{ji} & {\langle t_{r}|t_{r}\rangle }_{ji} \end{array}\right)$$

where the matrix elements represent the conditional probability for the *j^th^* residue to adopt the conformation *K* while the *i^th^* residue is in conformation *L*. Non-extended conformations in the left and right part of the Ramachandran plot are denoted as *t_l_* and *t_r_*. They can be expressed int terms of the Boltzmann factors:

(2) $$\langle L|{K\rangle }_{ji}=exp\left(\frac{G_{LK}+\delta G_{LK,ji}}{RT}\right)$$

where *G_L,i_* is the Gibbs energy of the conformation *L* in residue *j* and *δG_LK,ji_* is the NNI energy between residue *i* in state *K* and residue j in state L. 

The partition sum for the considered peptides can be written as:

(3) $$Z_{T}=p\cdot T_{12}\cdot T_{21}\cdot q_{1}$$

(4) $$Z_{P}=p\cdot T_{12}\cdot T_{23}\cdot T_{32}\cdot T_{21}\cdot q_{1}$$

where the subscripts *T* and *P* represents tetra- and pentapeptides, respectively. It should be noted that terminal residues are not taken into account. The vectors *q*_1_ and *p* are written as:

(5) $$q_{1}=\left(\begin{array}{c} exp\left(G_{pPII,1}/RT\right)\\ exp\left(G_{\beta ,1}/RT\right)\\ exp\left(G_{t_{l},1}/RT\right)\\ exp\left(G_{t_{r},1}/RT\right) \end{array}\right)$$

and

(6) p=(1,1,1,1)

A mistake in ref. [46] should be noted in this context: As *p* in Equation (6), the end vector *q_N_* (*N* denotes the *N^th^* residue) therein should have been written as unit vector. 

The main difference between the approach in ref. [46] and the current one is that the above formalism takes into account mutual interactions, while the one in the earlier paper solely accounts for a transfer from the N- to the C-terminal. To illustrate the point, let us consider a GxyG peptide. In our former approach the Boltzmann factors for the conformation of the y-residue depends on the conformations of the respective x-residue of the peptide. Hence, we solely considered transfer matrix elements of the type in the partition sum. The current approach adds conditional probabilities that account for the dependence of statistical weights of residue x conformations on the conformation adopted by residue y. The more restricted approach was chosen in ref. [46] to allow for an analytical approach to be used for the calculation of NNI-parameters from experimentally observed mole fractions. This differs from the below described optimization used for the current investigation. 

In order to obtain interaction parameters *δG_LK,ji_* we calculated mole fractions of individual residues and minimized the difference with experimentally determined values from an iterative least square fitting procedure for which *δG_LK,ji_* were used as free parameters. The Gibbs energies *G_K,i_* and *G_L,j_* were obtained from the experimentally determined mole fractions of residue conformations in GxG peptides [23,24,30,31]. To this end the Gibbs energies of pPII conformations were set to zero. Hence, all Gibbs energies in this study are in fact differences between the Gibbs energy of the considered conformation and pPII.

Individual mole fractions of residue conformations were calculated as:

(7) $$\chi_{L,j}=\frac{exp\left(\frac{\left[G_{L,j}+\sum_{K}\sum_{i}\left(G_{k,i}+\delta G_{LK,ji}\right)\delta_{j,j\pm 1}\right]}{RT}\right)}{\sum_{i}exp\left(\frac{\left[G_{L,j}+\sum_{K}\sum_{i}\left(G_{k,i}+\delta G_{LK,ji}\right)\delta_{j,j\pm 1}\right]}{RT}\right)}$$

where *δ_j*±1*,j_* is the Kronecker symbol. We would like to reiterate in this context that the terminal glycine residues of the considered GxyG and GxyzG peptides were not included in the analysis. Hence, for GxyzG, there are no *j* + 1 contribution for z (*j* = 3) and no *I* − 1 contribution for x (*j* = 1). 

Once NNIs were determined, the mole fraction of peptide conformations could be calculated, for tetra- and pentapeptides, respectively:

(8) $$\chi_{LK}=\frac{exp\left\{\left(G_{L,2}+G_{K,1}+\delta G_{LK,21}+\delta G_{KL,12}\right)/RT\right\}}{Z_{T}}$$

(9) $$\chi_{MLK}=\frac{exp\left\{\left(G_{M,3}+G_{L,2}+G_{K,1}+\delta G_{LK,21}+\delta G_{KL,12}+\delta G_{ML,23}+\delta G_{ML,32}\right)/RT\right\}}{Z_{P}}$$

Note that *M* is the superscript for considered conformations of the third residue (z) in the pentapeptide.

In order to further assess the influence of NNIs on the thermodynamics of the investigated peptides, we calculate the conformational entropy based on amino acid residue propensities in GxG and the isolated pair hypothesis with the conformational entropy in the presence of NNIs. For the former, we used the equation:

(10) $$S_{IPH}=-R\overset{N}{\underset{i=1}{\sum }}\left(\overset{4}{\underset{K=1}{\sum }}\left(\chi_{iK}ln\chi_{iK}\right)\right)$$

where

(11) $$\chi_{iK}=\frac{\exp\left(G_{iK}/RT\right)}{\sum_{K=1}^{4}\exp\left(G_{iK}/RT\right)}$$

is the population of the *K^th^* conformation of the *i^th^* residue. In the presence of NNIs, the entropy must be calculated by utilizing the *χ_LK_* and *χ_LKM_* values of Equations (8) and (9), for tetra- and pentapeptides, respectively:

(12) $$S_{NNI}^{tetra}=-R\overset{4}{\underset{K=1}{\sum }}\overset{4}{\underset{L=1}{\sum }}\left(\chi_{KL}ln\chi_{KL}\right)$$

(13) $$S_{NNI}^{penta}=-R\overset{4}{\underset{K=1}{\sum }}\overset{4}{\underset{L=1}{\sum }}\overset{4}{\underset{M=1}{\sum }}\left(\chi_{KLM}ln\chi_{KLM}\right)$$

The indices *K*, *L*, *M* are defined above.

## 3. Results

This section of the paper is organized as follows. First, we describe the results of the NMR-based conformational analysis of GAFG, GFAG and GKKG. Second, we compare mole fraction of corresponding residue conformations in GxG, GxyG and GxyzG peptides to illustrate the measurable effect of NNIs. Third, we describe our analysis of these data in terms of interaction parameters *δG_LK,ji_*.

### 3.1. Conformational Analysis

We augmented the already existing data sets obtained for a rather large number of tetra- and pentapeptides by GAFG and GFAG to further explore the influence of aromatic residues on their neighbors. In our recent study we showed that phenylalanine as neighbor has a tremendous and unexpected influence on the Ramachandran distribution of protonated D residues (and vice versa). Since alanine and aspartic acid residues stand out due to their extraordinary conformational sampling (pPII for A; β-strand, β-turn forming and asx-turns for D), we wondered how phenylalanine would affect alanine neighbors and vice versa. Therefore, we determined the values of the J-coupling constants listed in Materials and Methods for the tetrapeptides GAFG and GFAG. The results are listed in Table S1. It should be noted that we could not obtain reliable values for the ^1^J(H^N^,C’) constants of GAFG. We used the lsquarefit module of Matlab 2019b to perform the following fitting procedure. For each individual residue, we started with the statistical weights and basin coordinates obtained for GAG and GFG [23,31]. We allowed the statistical weights of pPII, β-strand, inverse γ, right- and left-handed helical conformations to vary in a fit to the coupling constants in Table S1. In a second step we optimized the fit by varying the basin coordinates of the considered conformations. Since ^1^J(H^N^,C’) values are not available for GAFG, we used the corresponding values obtained for GFG and GAG [23,31]. We did not vary the ψ-coordinates for this peptide. The thus obtained J-coupling parameters and the reduced χ_r_^2^ values of the fit are all listed in Table S1. Apparently, the reduced chi-square values indicate that the fitting reproduced the coupling constants of GFAG much better than the ones of GAFG. The unusually large values mostly reflect the differences between experimental and calculated ^3^J(H^N^,C’) values. For the F-residue the latter is lower than the lowest possible value that can be calculated with the corresponding Karplus equations. We observed similarly low values for arginine containing peptides [44]. Apparently, the empirical Karplus equation is less accurate for φ-values close to its minimum at −100°. We are not too concerned about the discrepancies regarding ^3^J(H^N^,C’) because our fits account at least qualitatively for the difference between the respective values of F and A (Table S1).

The obtained mole fractions are listed in Table 1 together with the corresponding mole fractions of the residues in GxG. The Ramachandran plots of the peptides are shown in Figure S1. The respective positions of the basins are listed in Table S2. Interestingly, phenylalanine has a very limited influence on alanine when positioned downstream, while it stabilizes β-strand over pPII as expected when positioned upstream. Alanine as downstream neighbor of phenylalanine stabilizes pPII over β while it produces the opposite effect when positioned upstream. In addition, we observed a slight increase of left-handed helical conformations for all residues. This is a little bit surprising, but we found that omitting this conformation from the list led to a deterioration of the overall quality of the fit.

We also measured and analyzed the J-coupling constants of cationic GKKG. The experimental and calculated J-coupling constants are listed in Table S3. The mole fractions of the considered conformations are listed in Table S4. Below, we will discuss these results only in passing, because a limited number of coupling constants were used for the reference system GKG. The Ramachandran plots of the K-residues in these peptides are shown in Figure S2. Interestingly, the plot for the C-terminal K-residue is very similar to the GKG Ramachandran, which indicates a weak influence from the upstream neighbor. On the contrary, the downstream neighbor shifts the N-terminal distribution much towards pPII. 

### 3.2. Homopeptides

**Figure 2:**
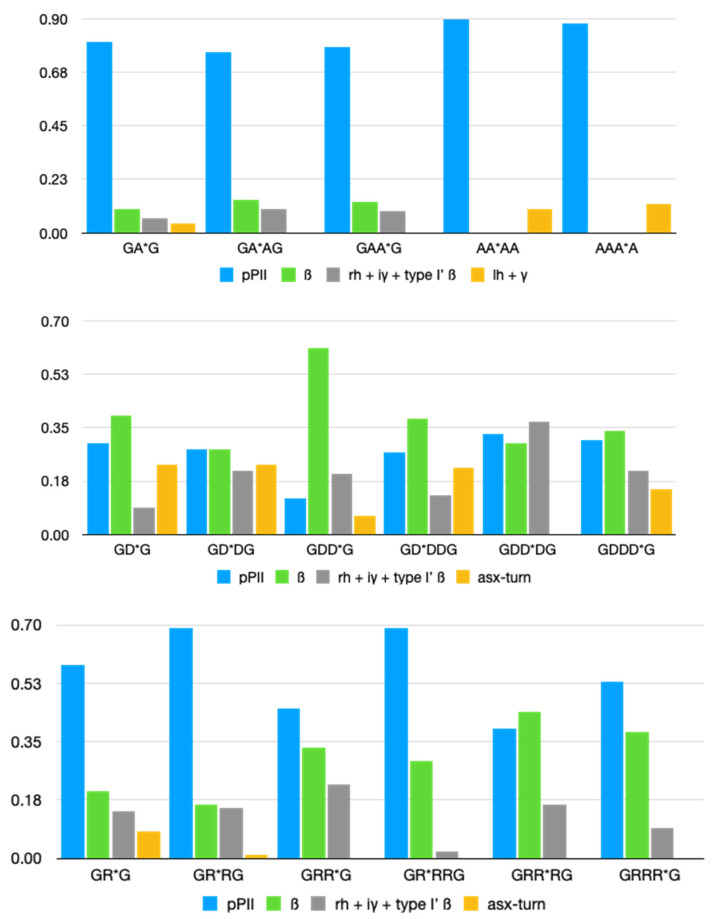
Statistical weights of the indicated residue conformations in the Ramachandran distribution of alanine, aspartic acid and arginine-based tetra- and penta-homopeptides. The respective residue for which the statistical weights are plotted is labeled with an asterisk. The respective values have been taken from refs. [44,45].

Figure 2 compares the statistical weights of pPII, β-strand, the combined mole fractions of inverse γ-turns, type I/II’ *(i* + 2) β-turn and right-handed helical as well as the combination of asx-turns, left-handed helical and γ-turns for homopeptide segments containing alanine, protonated aspartic acid [45] and arginine [44]. The numerical values are listed in Table S6. 

For alanine, the differences between the statistical weights are nearly within their limits of accuracy, but it seems that the combined influence of alanine on both sides slightly enhances the pPII propensity of the central alanines in AAAA, in agreement with predictions from MD simulations [22]. For aspartic acid residues containing peptides, three aspects of NNI caused changes which are most remarkable. First, the downstream D-residue in GDDG is heavily affected by its neighbor in that the latter causes a significant stabilization of β-strand at the expense of pPII and a slight stabilization of this residue’s peculiar population of type I/II’ β-turn forming conformation. Second, the combined influence from both neighbors on the central residue in GDDDG causes a nearly canonical random coil supporting distribution with the sole difference of the right-handed helix basin being replaced by one associated with conformations found at the *i* + 2 position of type I/II’ β-turns. Asx-turns are no longer sampled by this residue. Third, NNIs in all D-peptides favor type I/II’ β-turn forming structures over asx-turns. The presence of the former in coil library-based Ramachandran plots of aspartic acid residues is noteworthy in this context [45]. 

Interestingly, NNIs in the investigated arginine-based peptides also cause some sort of conformational randomization. The Ramachandran plot of GRG is clearly dominated by pPII sampling, which is mostly maintained for the most upstream R-residue in GRRG and GRRRG. However, the Ramachandran plots of the second and third arginine residues look vastly different, in that they are indicative of a significant β-strand stabilization. Compared with D-Ramachandran plots, the distributions are indicative of a larger sampling of the basins in the upper left quadrant of the Ramachandran plot. 

We used our thermodynamic model outlined in the theoretical section of this paper to determine the NNI parameters by adopting the following fitting procedure. Our analysis was based on the assumption that NNIs are dominated by interaction between pPII and β-strand conformations of adjacent residues. Hence, we calculated mole fractions as a function of the interaction parameters *δG_pPIIβ,ji_* and *δG_βpPII,ji_* for all residues. Respective chi-square functions reflecting the differences between calculated and experimentally obtained mole fractions were minimized. In a second step, the chi-square values were minimized further by considering interactions between helical/turn conformations and pPII/β-strand. Some of the obtained interaction parameters carry a rather large statistical uncertainty due to very flat chi-square functions. In line with Schweitzer-Stenner and Toal we generally did not consider pPII-pPII and β-β interactions since the experimental ones are clearly indicative of pPII-β strand correlation [46]. The intrinsic Gibbs energy differences were calculated from the mole fractions reported for GxG peptides [23,24,30,31,51].

The analysis of interactions involving the central residue in GRRRG and GDDDG is complicated by the fact that it is influenced by two neighbors. Here, we assumed that the parameters describing the influence of the first on the second guest residue can be taken from the corresponding GxxG parameters for the above D- and R-containing peptides. We then minimized the difference between experiment-based and calculated mole fractions by varying the parameters for the interaction between the second and third residue. In addition, we still varied the parameters reflecting the influence of the central residue on its upstream neighbor. In an ideal case, namely the absence of any second neighbor interactions, this step would be superfluous. Its necessity indicates that these interactions are in fact present. They are not explicitly considered in our model.

It should be mentioned that we did not carry out this analysis of the alanine peptides because NNI-induced changes are small. The observed changes of statistical weights indicate some type of pPII-pPII cooperativity, in agreement with computational predictions [22,52]. 

**Figure 3:**
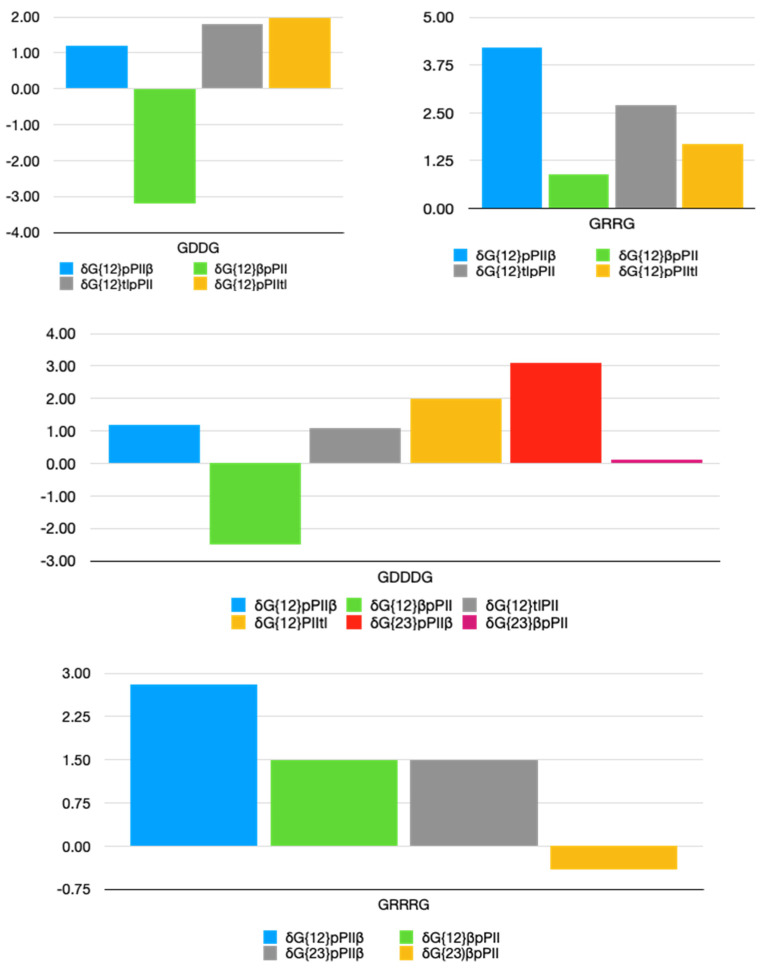
Bar plots of nearest neighbor interaction Gibbs energy parameters (in units of kJ/mol) for aspartic acid and arginine residues in GDDG, GDDDG, GRRG and GRRRG. For these diagrams we combined the interaction Gibbs energies reflecting the influence of the *i^th^* residue adopting conformation *K* on the energetics of conformation *L* of residue *j* and the (influence of the *j^th^* residue adopting conformation *L* on the energetics of conformation *K* of residue *i*) to display the total contribution of NNIs to a pair of residues *j* and *i* adopting conformations LK, respectively.

The obtained values for all pPII-β and β-pPII interactions are displayed in Figure 3. Note that we added up the values of interaction parameters that affect the same state of residue dimers, e.g., $\delta G_{pPII\beta }^{\left\{12\right\}}=\delta G_{pPII\beta ,12}+\delta G_{pPII\beta ,21}$, where {12} just indicates the residues constituting the considered dimer. A complete set of all individual parameters is listed Table S7. Positive and negative NNIs indicate a stabilization and a destabilization of pPII-β and β-pPII pairs, respectively. To assess the significance of obtained NNI parameters, the thermal energy (~2.5 kJ/mol) should be used as a benchmark. Generally, only values of more than one half of this value are considered as significant.

The charts in Figure 3 indicate that NNIs are rather different for pairs of aspartic acid and arginine residues. For DD, the pPII-β state is slightly stabilized while the β-pPII state is significantly destabilized. This leads to the observed increase of the β-strand population of the second D-residue. t_l_-pPII and pPII-t_l_ dimers are both stabilized. However, the NNI-parameters for the latter are subject to pronounced statistical errors (Table S7). In GRRG, however, pPII-β, β-pPII, t_l_-pPII and pPII-t_l_ pairs are all stabilized by NNIs. The cooperativity between pPII and β conformations leads to balanced pPII-β distributions in the Ramachandran plots of GRRG. NNI patterns are different in the corresponding pentapeptides. In GDDDG, the D1D2 dimer interactions resemble the ones observed for GDDG, while pPII-β conformations of D2D3 are clearly stabilized. Hence, NNIs between D1 and D2 are in part neutralized by interactions between D2 and D3. Interactions involving helical and turn conformations of D2 and D3 were not considered due to their large statistical errors. In GRRRG, pPII-β and β-pPII dimers are all stabilized. 

It is noteworthy in this context that the conformational analysis of GKKG reveals a significant positive pPII-β strand interaction between the first and second K-residue in GKKG (~3 kJ/mol). This value is indicative of a strong stabilization of pPII-β pairs, which is in line with our results for GRRG. Thus, the results obtained for the positively charged homodimers point in the same direction. 

### 3.3. Heteropeptides

**Figure 4:**
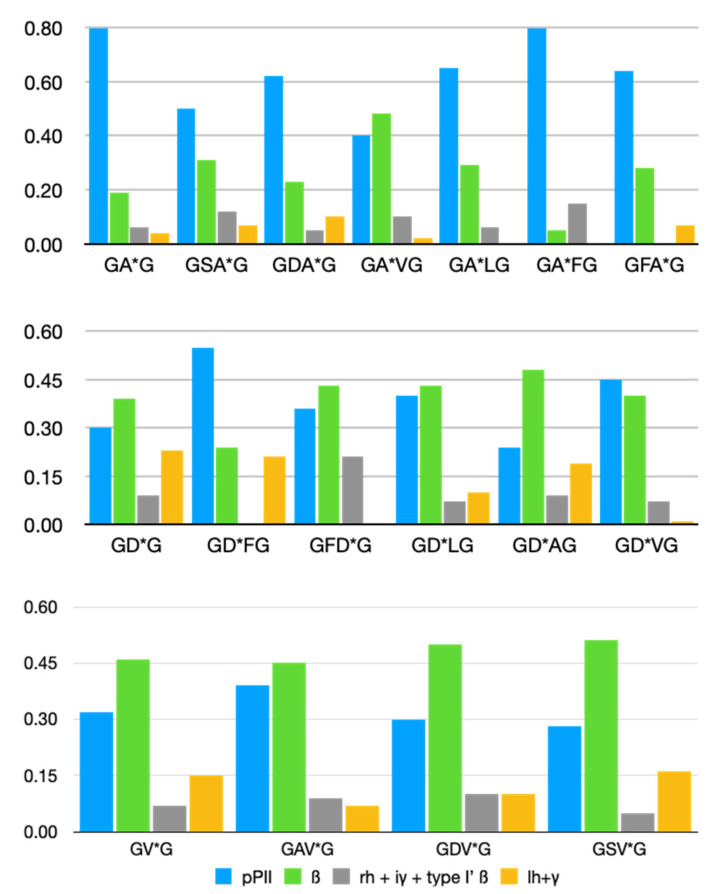
Statistical weights of the indicated residue conformations in the Ramachandran distribution of alanine, aspartic acid and valine based hetero-tetrapetides. The respective residue for which the statistical weights are plotted is labeled with an asterisk. The respective values have been taken from ref. [53].

Figure 4 exhibits the statistical weights (mole fractions) of alanine, aspartic acid and valine without and with different neighbors. The data were taken from ref. [43], with the exception of DF and FD that can be found in ref. [45]. Values for AF and FA are reported in this paper (Table 1). 

Apparently, the pPII and β-strand values of alanine and aspartic acid are substantially changed by neighbors. For alanine, NNIs are particularly prominent for V, S and to a lesser extent for F (FA). Aspartic acid is significantly affected by all its investigated neighbors. On the contrary, valine is much less influenced by its neighbors.

**Figure 5:**
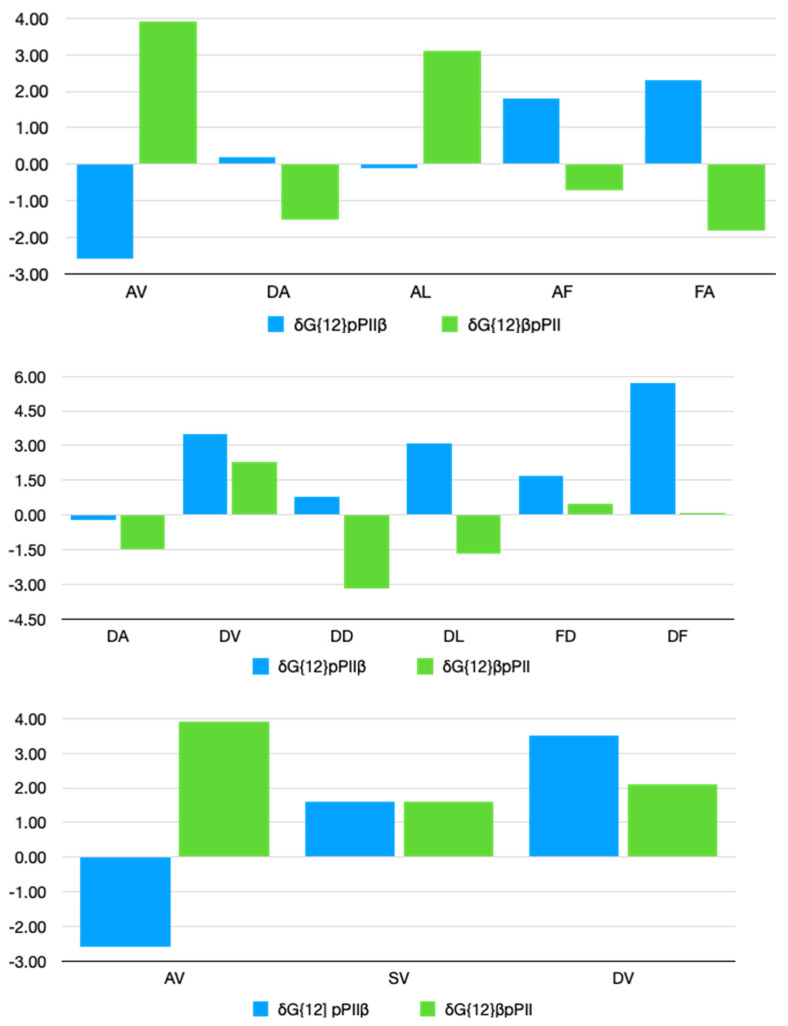
Nearest neighbor interaction Gibbs energy parameters (in units of kJ/mol) for the indicated residue pairs in tetrapeptides as obtained from the optimization procedure described in the text. For these diagrams we combined the interaction Gibbs energies reflecting the influence of the *i^th^* residue adopting conformation *K* on the energetics of conformation *L* of residue *j* and the (influence of the *j^th^* residue adopting conformation *L* on the energetics of conformation *K* of residue *i*) to display the total contribution of NNIs to a pair of residues *j* and *i* adopting conformations LK, respectively.

Figure 5 shows the pPII-β interaction parameters for residue pairs in tetrapeptides. They are listed in Table S8, together with some statistically meaningful pPII-turn/helix interactions. Interaction parameters affecting the same dimer sequence were again added up (*vide supra*). The figure is organized in a way that it shows the influence of alanine, aspartic acid and valine on a set of different neighbors and vice versa. 

For the alanine-containing series, pPII-β sequences are mostly destabilized by V but stabilized by F irrespective of its position. β-pPII conformations are stabilized for AV and AL but destabilized by F in both AF and FA dimers. This leads to the above described rather opposite influence of the up- and downstream F on A. The parameters in Table S8 reveal that the interaction parameters in Figure 5 predominantly reflect the influence of neighbors on alanine. The NNI parameters of D-containing tetrapeptides suggest that pPII-β dimers are stabilized by all of the considered neighbors, though to a different extent. F, V and L are very effective in this regard. A, D and L as neighbors destabilize β-pPII, while V stabilizes it moderately. For V-containing peptides, pPII-β and β-pPII dimers are stabilized, pPII-β of GAVG being the sole exception. 

Taken together, the results of our analysis suggest a rather prominent role of pPII-β interactions. If positive it stabilizes pPII-β pairs, while it stabilizes pPII and/or β pairs if negative. pPII-β dimers are stabilized for the majority of the considered pairs, while the NNI effects on β-pPII conformations can be stabilizing or destabilizing, depending on the type and the sequence of the two residues. While alanine has a limited effect on neighbors (F being the very significant exception), V is not much influenced by it. 

## 4. Discussion

Our analysis of experimentally determined conformational propensities of amino acid residues in tetra- and pentapeptides was based on a statistical mechanics model that assigned changes of intrinsic propensities (i.e., populations of basins in the absence of any NNIs) solely to cooperative effects between neighboring residues. The term ‘cooperativity’ means that changes of propensities depend on the conformation the neighbors of a residue adopt. Earlier obtained anti-correlations between pPII and β-strand propensities of residues in tetrapeptides suggest that NNIs are to a significant extent cooperative in nature [46]. In the presence of cooperative (conformation-dependent) NNIs, the statistical weights of amino acid conformations have to be replaced by conditional probabilities. In this study we did that by utilizing an approach that resembles a one-dimensional Ising model. While the latter considers only two states per unit (i.e., magnetic moments associated with magnetic spin quantum number ½ and –½), our model contains four states per residue. However, our results strongly suggest that NNIs between pPII and β-strand are dominant at least in short peptides. The Ising model is generally used to describe either ferro- or anti-ferromagnetism. In other words, the system is either cooperative or anti-cooperative. In our model we allow for combinations of cooperative and anti-cooperative NNIs. Combinations of the two depend very much on the amino acid residue sequence. A stochastic combination of anti-ferromagnetism and ferromagnetism is generally considered a spin glass [54]. While spin-glass-like behavior has long been identified as part of the protein folding process, [55] its relevance for an understanding of unfolded peptides and proteins still has to be explored. In what follows, we will focus on two aspects of the obtained NNIs, namely the stabilization of sequences with mixed conformations and the implications for conformational entropy.

### 4.1. Peptide Conformations

**Figure 6:**
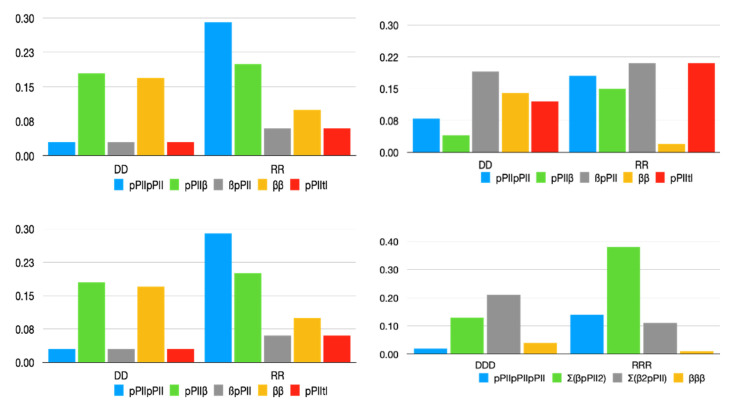
Statistical weights (mole fractions) of conformations of GxxG and GxxxG peptides in the absence (**left**) and presence of nearest neighbor interactions (**right**). The Σ-sign indicates a summation over all conformations with the indicated number of residue conformations.

In the absence of NNIs, the statistical weight of individual peptide conformations would just be the product of mole fractions of individual residues. In the presence of NNIs, the probability of a residue to adopt a certain conformation depends on the conformation of its neighbors. Hence, one has to use Equation (6) to calculate peptide conformations. Figure 6 compares the statistical weight of peptide conformations adopted by GxxG and GxxxG peptides calculated with and without NNIs. For the former, we used the mole fractions reported for the corresponding GxG peptides. For GDDG, NNIs increase the population of β-pPII and pPII-tl populations. NNIs in GRRG stabilize pPII-β, β-pPII and pPII-tl conformations by concomitantly destabilizing the all β-strand and pPII states, respectively. Thus, NNIs cause a larger degree of equipartition among the different conformational sequences. It should be noted that t_l_ represents mostly type I/II’ β-turn supporting conformation for GDDG while the respective basins of GRRG lie in the right-handed helical region. 

For GxxxG peptides, Figure 6 compares the fraction of all-pPII, all-β-strand and the combined fraction of conformations with 2 pPII/1β and pPII/2β, respectively. In GDDDG, NNIs clearly enhance the population of conformations with a mixture of pPII and β, in part at the expense of turn-supporting conformations not displayed in Figure 6. For GRRRG, we obtained a significant enhancement of conformations with two pPII and one β-strand conformation at the expense of conformations dominated by β-strand and of sequences with asx-turns (not displayed). It is noteworthy that βpPIIpPII contributes most to the 2pPII1β ensemble (~0.2), followed by pPIIβpPII (~0.14). 

**Figure 7:**
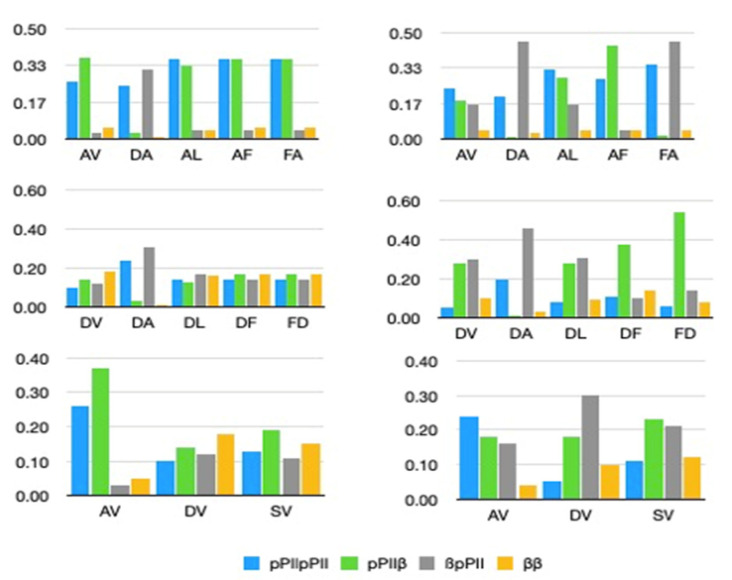
Statistical weights (mole fractions) of conformations of GxyG peptides in the absence (**left**) and presence of nearest neighbor interactions (**right**).

Figure 7 compares the statistical weight of GxyG peptide conformations for series with alanine, aspartic acid and valine neighbors. It is noteworthy that NNIs enhance the β-pPII population in a majority of these peptides, namely GDAG, GFAG, GDVG, GDLG, GSVG, and GDLG. pPII-β populations are enhanced for GFAG, GDFG and GFDG. For the alanine series the substantial NNI-induced increase of β-pPII in the distribution of GDAG and GFAG is particularly remarkable. 

Taken together, our analysis confirms the expectation that NNIs increase the population of mixed pPII-β conformations. The increase of pPII-t_l_ populations in GDDG and GRRG is noteworthy but not reproduced in the investigated pentapeptides, where conformational entropy distributes residues adopting a t_l_- or even a t_r_ conformation over many different sequences with residues adopting pPII and β-strand conformations. On one side, the increased populations of mixed structures seem to produce more random distributions for a single residue. On the other side, the correlation between the conformational dynamics of residues seems to reduce randomness. This notion is tested further in the subsequent section. 

### 4.2. NNIs and Entropy

**Figure 8:**
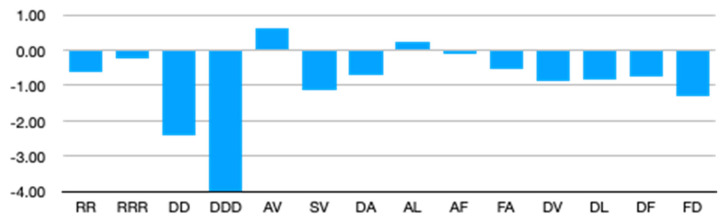
Differences between the Gibbs energy contributions of conformational entropies (in kJ/mol) at 300 K associated with the indicated peptide segments in the absence and presence of nearest neighbor interactions. Negative values indicate a lower entropy for the indicated peptide conformations in the presence of NNIs.

We calculated the conformational entropy using the mole fraction of the considered residues in GxG (absence of NNIs) and the mole fractions calculated for peptide conformations in the presence of NNIs. It should be noted that the calculated values should not be taken as absolute since we do not consider the population of individual basins in the Ramachandran plot. However, since the widths of respective distributions are not very different, the difference between corresponding entropy values provides a meaningful measure of the NNI-induced entropy changes. The room temperature Gibbs energy values corresponding to the differences between these entropies are displayed in Figure 8. The entropy values were calculated using Equations (8) and (9). With a few exceptions (GAVG and GALG), NNIs reduce the conformational entropy. Difference values of GxyG peptides lie mostly in the 10^2^ J/mol range at 300 K, but some values are larger than 1 kJ/mol (GSVG and GFDG). For the two investigated pentapeptides, the difference is more pronounced (2.4 kJ/mol for GRRRG and 4.0 kJ/mol for GDDDG). Even if values for corresponding heteropeptides might be lower, these values indicate that the entropy reduction for longer polypeptides could be substantial. Our results show that generally Ramachandran plots of individual residues cannot be used to estimate conformational entropies since this might lead to a substantial overestimation. MD results that seem to confirm the additivity of conformational entropies should be considered with great caution [56], since recent MD simulations of GRRG and GRRRG with a CHARMM36m force field and a TIP3P water model did not yield any substantial nearest neighbor effects, in clear contrast to experimental data [44]. This was attributed to the inability of the used water model to account for cooperative interactions between the hydration shells of adjacent residues. However, the results of Zaman et al. strongly suggest that the choice of the force field also matters [40]. 

While NNIs seem to randomize Ramachandran distributions of residues by reducing propensity differences between pPII and β-strand and by increasing right-handed helical or turn-supporting conformations in some (homopeptide) cases, they actually reduce the conformational entropy of peptides. The analyses of unfolded proteins by Baxa et al. suggest that this view can be generalized [53]. Assessing the entropy of unfolded states is a relevant topic in the context of intrinsically disordered proteins owing to their involvement in binding and allosteric processes which involve either disorder→order or order→disorder transitions [6,13,57]. 

The question arises how to estimate the conformational entropies of entire unfolded proteins and longer polypeptides. In principle, this task could be accomplished by molecular dynamics calculations [58,59,60]. However, conformational sampling and the inadequacy of current forcefields for a modelling of disordered proteins might produce serious obstacles [40,44,51,61]. Moreover, Baxa et al. showed that the use of covariance matrices derived from MD simulations actually leads to an overestimation of the conformational entropy of unfolded proteins [53]. These authors presented the combined use of coil library data and MD simulations as a remedy which worked quite well. The differences between conformational distributions of amino acid residues in short peptides and coil libraries might indicate that the latter do not fully represent residues in denatured or unfolded proteins [31,32]. 

What are the *pros* and *cons* of using short model peptides to elucidate NNIs? Some advantages are obvious. First of all, one can be assured that the determined conformational distributions are not subject to outside constraints such as non-local interactions and intramolecular hydrogen bonding. Second, residue-specific interactions can be identified and quantified, as done in this paper. This provides some fundamental insight into the statistical thermodynamics of statistical coils in general and about how NNIs affect conformational distributions in particular. However, in order to extrapolate the findings achieved with model peptides to longer polypeptides and even proteins, one might need data for a larger number of nearest neighbor combinations. Obviously, investigating even a complete set of e.g., GxyG peptides with the techniques used by Toal et al. [43] appears as an impossible task. A sensible solution might be to confine choices to representative amino acid residues. Alanine should be considered as a category of its own. Aspartic acid could represent all residues with side chains capable of forming hydrogen bonds [24]. Phenylalanine is certainly a suitable representative of aromatic residues. Valine, which has a major impact on some neighbors, could represent residues with branched side chains such as isoleucine and even threonine [25,32]. Arginine and/or glutamic acid could represent charged side chains. Proline and glycine are in their respective own categories. Confining future investigations to combinations of these residues is a more doable task owing to the already available data. 

Some unanswered conceptual questions deserve to be emphasized. Whereas our analysis has revealed that sequences with mixed pPII-β and β-pPII conformations might be frequently stabilized over homogeneous sequences containing residues with the same conformation, the question arises about the persistence length of protein segments with distinct conformational sequences. Answering this question is relevant because it determines to what extent the conformational entropy of a statistical coil deviates from the sum of entropies of individual residues. Moreover, it is unclear to what extent NNIs between pPII/β- and right-handed helical states as well as between right-helical states become relevant with increasing length of an oligopeptide. The canonical Zimm–Bragg model suggests just that [62]. As a matter of fact, the validity of this model actually requires the break-down of the isolated pair hypothesis, since it assumes that the probability for a residue to adopt a helical conformation depends on whether its neighbor is in a coil or helical state. It is therefore obvious that some longer oligopeptides must be investigated as well in order to explore the admixture of helical conformations to individual Ramachandran plots. An early structural analysis of salmon calcitonin and Aβ_1–28_ shows how this goal could be achieved [63].

## 5. Summary

Nearest neighbor interactions in unfolded and denatured proteins were a rather popular subject for some time after NMR data provided hints for their existence [35,64]. The work of the Sosnick group provided compelling evidence for the notion that NNIs must be considered in order to account for conformational distributions of unfolded proteins [65]. Most analyses of NNIve thus far been based on coil library distributions of amino acid residues [17,66]. However, it is unclear whether these distributions fully represent the statistical coil of unfolded and denatured proteins in good solvents [31]. An alternative approach utilizes short peptides for which it is easier to obtain quantitative information about NNI energies [41,42,43,44,45]. The analysis of experimentally observed amino acid residue propensities in tetra- and pentapeptides presented in this paper provide compelling evidence for the notion that conformational motions of neighboring residues are correlated. We showed that NNIs between adjacent amino acid residues are cooperative/anticooperative in nature as well as residue and sequence dependent. Gibbs interaction energies vary over a range that exceeds the thermal energy at room temperature. 

## Figures and Tables

**Table 1:** List of statistical weights of conformations constituting the Ramachandran plots of the indicated peptides. The values for GAG and GFG have been taken from refs. [23,31].

Conformation	GAG	GAFG	GFAG	GFG	GFAG	GAFG
**pPII**	0.8	0.8	0.64	0.45	0.61	0.37
**β-strand**	0.1	0.05	0.27	0.45	0.32	0.57
**iγ/type II β**	0.03	0	0	0	0	0
**rh**	0.03	0	0	0.05	0	0
**lh**	0.04	0.15	0.07	0.05	0.06	0.06

## Data Availability

All relevant NNI interaction parameters are presented in the paper or in the Supporting Information. Additionally, published data sets have been used and properly referenced. Text files of the Ramachandran plots of GAG, GFG, GAFG, GFAG, GKG, and GKKG were deposited at Schweitzer-Stenner, Reinhard (2022), “Ramachandran Plots of Amino Acid Residues”, Mendeley Data, V1, https://doi.org/10.17632/7fwh6j8rn8.1, accessed on 28 April.

## Supplementary Materials

The following supporting information can be downloaded at: https://www.mdpi.com/article/10.3390/biom12050684/s1, Figure S1: Ramachandran plots of alanine and phenylalanine containing peptides; Figure S2: Ramachandran plots of lysine containing peptides. Table S1: Experimental and calculated J-coupling constants of GAFG and GFAG; Table S2: Positions of Gaussian sub-distributions of alanine and phenylalanine containing tetrapeptides; Table S3: Experimental and calculated J-coupling constants of GKKG; Table S4: Statistical weights of sub-distributions of GKG and GKKG; Table S5: Positions of Gaussian sub-distributions of GKG and GKKG; Table S6: Statistical weight of peptide conformations in homopeptide sequences; Table S7: Nearest neighbor coupling parameter values of homopeptides; Table S8: Nearest neighbor coupling parameter values of heteropeptides.
