# Supplementary material for: Randomizing of Oligopeptide Conformations by Nearest Neighbor Interactions between Amino Acid Residues
Source: Biomolecules. 2022 May 11;12(5):684. doi: 10.3390/biom12050684 (PMC9138747; doi:10.3390/biom12050684)
Supplement: Supplementary file 1 [file biomolecules-12-00684-s001.zip › biomolecules-1660874 supplementary.pdf]

Randomization of Oligopeptide Conformations by Nearest Neighbor Interactions between Amino Acid Residues

Reinhard Schweitzer-Stenner,[1] Harald Schwalbe[2] and Bridget Milorey[1]*

[1]Department of Chemistry, Drexel University, Philadelphia, PA 19104, USA and [2]Institut für Organische Chemie und Chemische Biologie, Johann Wolfgang Goethe Universität, 60438 Frankfurt, Germany

Supporting Information

*Corresponding author: Reinhard Schweitzer-Stenner, Phone: 215-895-2268; Email: rs344@drexel.edu

**J-coupling constants [Hz]**	G**A**FG	GA**F**G	G**F**AG	GF**A**G
**$^3J(H^NH^\alpha)$**	Exp: 6.24<br>Gauss: 6.08	Exp: 7.67<br>Gauss: 7.78	Exp: 6.92<br>Gauss: 6.98	Exp: 7.02<br>theo: 6.81
**$^3J(H^NC')$**	Exp: 1.21<br>Gauss: 1.04	Exp: 0.33<br>Gauss: 0.76	Exp: 1.01<br>Gauss: 0.97	Exp: 1.14<br>Gauss: 1.03
**$^3J(H^\alpha C')$**	Exp: 2.2<br>Gauss:2.4<br>2	Exp: 2.48<br>Gauss: 2.43	Exp: 2.22<br>Gauss: 2.24	Exp: 2.08<br>Gauss: 2.24
**$^3J(H^NH^\beta)$**	Exp: 1.94<br>Gauss: 1.96	Exp: 1.26<br>Gauss: 1.38	Exp: 1.81<br>Gauss: 1.65	Exp: 2.06<br>Gauss:1.63
**$^1J(H^NH^\alpha)$**	-		Exp: 11.7<br>Gauss:<br>11.63	Exp: 11.3<br>Gauss: 11.4
**χ_r^2**	5.49	8.17	0.2	1.67

Table S1: Experimental and calculated (Gauss) J-coupling constants of the indicated residues of the cationic tetrapeptides GAFG and GFAG. The respective residues are typed in bold in the header. The reduced chi-square value of the fits are listed at the bottom of the table.

**Basins**	GAG	G**A**FG	GF**AG**	GFG	G**F**AG	GA**F**G
**pPII**	−72°,150°	−73°,150°	−73°,150°	−77°,155°	−72°,152°	−74°,152°
**β-strand**	−115°,150°	−120°,150°	−115°,150°	−115°,155°	−120°,150°	−115°,150°
**iγ**	−60°,60°	−60°,60°	−60°,60°	−80°,60°	−80°,60°	−80°,60°
**rh**	−60°,−30°	−60°,−30°	−60°,−30°	−65°,−20°	−65°,−30°	−65°,−30°
**lh/γ**	60°,−60°	70°,50°	70°,50°	80°,20°	75°, 60^0	80°,60°

Table S2: Positions of Gaussian sub-distributions representing basins in the Ramachandran plot of the indicated peptides

J-coupling constants [Hz]	G**K**KG	GK**K**G
**$^3J(H^NH^\alpha)$**	Exp: 6.42<br>Gauss: 6.47	Exp: 7.05<br>Gauss: 6.93
**$^3J(H^NC')$**	Exp: 0.91<br>Gauss: 0.96	Exp: 1.11<br>Gauss: 1.96
**$^3J(H^\alpha C')$**	Exp: 1.95<br>Gauss:2.0	Exp: 1.75<br>Gauss: 1.97
**$^3J(H^NH^\beta)$**	Exp: 1.83<br>Gauss: 1.96	Exp: 1.54<br>Gauss: 1.38
**$^1J(H^NH^\alpha)$**	Exp: 11.79<br>Gauss:<br>11.72	Exp:11.60<br>Gauss: 11.60
**χ_r^2**	0.25	0.72

Table S3: Experimental and calculated (Gauss) J-coupling constants of the indicated residues of the cationic tetrapeptide GKKG. The respective residues are typed in bold in the header. The reduced chi-square value of the fits are listed at the bottom of the table.

Conformation	GK<br>G	G**K**K<br>G	GK**K**<br>G
**pPII**	0.5	0.81	0.54
**β-strand**	0.41	0.156	0.46
**iγ/type II β**	0.0	0.04	0
**rh**	0.09	0	0
**lh/γ**	0.0	0.14	0

Table S4: List of statistical weights of conformations constituting the Ramachandran plots of the indicated peptides.

Basins	GKG	G**K**KG	GK**KG**
**pPII**	−66°,150°	−74°,155°	−66°,150°
**β-strand**	−115°,145°	−115°,155°	−115°,150°
**iγ**		−60°,30°	−20°,20°
**rh**	−65°,−30°	−65°,−30°	−65°,−30°
**lh/γ**		60°,30°	20°,−50°

Table S5: Positions of Gaussian sub-distributions representing basins in the Ramachandranplot of the indicated peptides.

	pPII	ß-strand	rh+iγ+type I' ß	lh+γ
**GA*G**	0.80	0.10	0.06	0.04
**GA*AG**	0.76	0.14	0.10	0.00
**GAA*G**	0.78	0.13	0.09	0.00
**AA*AA**	0.90	0.00	0.00	0.10
**AAA*A**	0.88	0.00	0.00	0.12

	pPII	ß-strand	rh+iγ+type I' ß	asx-turn
**GD*G**	0.30	0.39	0.09	0.23
**GD*DG**	0.28	0.28	0.21	0.23
**GDD*G**	0.12	0.61	0.20	0.06
**GD*DDG**	0.27	0.38	0.13	0.22
**GDD*DG**	0.33	0.30	0.37	0.00
**GDDD*G**	0.31	0.34	0.21	0.15

	pPII	ß-strand	rh+iγ+type I' ß	asx-turn
**GR*G**	0.58	0.20	0.14	0.08
**GR*RG**	0.69	0.16	0.15	0.01
**GRR*G**	0.45	0.33	0.22	0.00
**GR*RRG**	0.69	0.29	0.02	0.00
**GRR*RG**	0.39	0.44	0.16	0.00
**GRRR*G**	0.53	0.38	0.09	0.00

Table S6: Statistical weight of the indicated conformations in the Ramachandran plots of alanine, aspartic acid and arginine based homopeptide sequences. Values were taken from ref. [43–45,51]

	$\delta G_{pPII\beta,12}$	$\delta G_{\beta pPII,12}$	$\delta G_{pPII\beta,21}$	$\delta G_{\beta pPII,21}$
**GRRG**	2.2±0.5	1.0±0.3	−0.1±0.3	2.0±0.3

	$\delta G_{tlpPII,12}$	$\delta G_{tlpPII,21}$
**GRRG**	2.7±1.0	1.7±0.8

	$\delta G_{pPII\beta,12}$	$\delta G_{\beta pPII,12}$	$\delta G_{pPII\beta,23}$	$\delta G_{\beta pPII,23}$	$\delta G_{pPII\beta,32}$	$\delta G_{\beta pPII,32}$
**GRRRG**	0.8±0.7	1.6±0.6	0.1±0.6	0.7±0.6	−1.1±0.7	1.4±0.6

	$\delta G_{pPII\beta,12}$	$\delta G_{\beta pPII,12}$	$\delta G_{pPII\beta,21}$	$\delta G_{\beta pPII,21}$
**GDDG**	0.2±0.5	−0.5±0.5	−2.7±0.6	1±0.6

	$\delta G_{tlpPII,12}$	$\delta G_{tlpPII,21}$
**GDDG**	1.8±1.0	2.0±0.8

	$\delta G_{pPII\beta,12}$	$\delta G_{\beta pPII,12}$	$\delta G_{pPII\beta,23}$	$\delta G_{\beta pPII,23}$	$\delta G_{pPII\beta,32}$	$\delta G_{\beta pPII,32}$
**GDDDG**	0.2±0.5	0.2±0.5	3.8±0.5	0.7±0.4	−0.6±0.5	−0.7±0.4

	$\delta G_{tlpPII,12}$	$\delta G_{tlpPII,21}$
**GDDDG**	1.1±1.0	2.0±0.8

Table S7: Parameters reflecting nearest neighbor interactions in the indicated peptides. In addition to pPII-β NNIs we list some interaction parameters involving right-handed helical and turn conformations for which the statistical error is not larger than the value itself.

x,y	$\delta G_{pPII\beta,12}$	$\delta G_{\beta pPII,12}$	$\delta G_{pPII\beta,21}$	$\delta G_{\beta pPII,21}$	$\delta G_{pPIItl,12}$	$\delta G_{tlpPII,12}$	$\delta G_{pPIItl,21}$	$\delta G_{tlpPII,21}$
**AV**	−2.9±0.5	3.2±0.5	0.7±0.3	0.3±0.5	1.3±0.6	1.7±1.0	-	-
**DA**	−0.1±0.4	1.1±0.3	−2.6±0.4	0.3±0.5	-	-	−2.0±1.0	-
**AL**	−0.3±0.5	3±0.5	0.1±0.5	0.2±0.4	-	-	-	-
**AF**	2±0.5	0.2±0.3	−0.9±0.4	0.2±0.5	4.0±1.0	-	−1.4±1.0	-
**FA**	1.7±0.5	0.2±0.4	−3±0.7	0.5±0.5	-	−2.0±1.0	-	-

x,y	$\delta G_{pPII\beta,12}$	$\delta G_{\beta pPII,12}$	$\delta G_{(pPII\beta,21)}$	$\delta G_{\beta pPII,21}$	$\delta G_{pPIItl,12}$	$\delta G_{tlpPII,12}$	$\delta G_{pPIItl,21}$	$\delta G_{tlpPII,21}$
**DA**	−0.1±0.4	1.1±0.3	−2.6±0.4	0.3±0.5	-	-	−2.0±1.0	-
**DV**	3.1±0.3	2.2±0.3	0.1±0.5	0.4±0.3	-	-	-	-
**DD**	−0.2±0.5	−0.5±0.5	−2.7±0.6	1.0±0.7	-	1.8±1.0	-	2.0±1.0
**DL**	2.7±0.3	−2.3±0.3	0.6±0.5	0.4±0.5	-	-	-	-
**FD**	6.0±0.4	0.7±0.4	−0.6±0.5	−0.3±0.6	-	1.5±1.0	-	1.7±1.0
**DF**	1.6±0.3	1.5±0.3	−1±0.4	0.1±0.5	-	-	-	-

x,y	$\delta G_{pPII\beta,12}$	$\delta G_{\beta pPII,12}$	$\delta G_{pPII\beta,21}$	$\delta G_{\beta pPII,21}$	$\delta G_{pPIItl,12}$	$\delta G_{tlpPII,12}$	$\delta G_{pPIItl,21}$	$\delta G_{tlpPII,21}$
**AV**	−2.9±0.5	3.2±0.5	0.7±0.3	0.3±0.5	1.3±0.6	1.7±1.0	-	-
**SV**	1.5±0.4	2.1±0.4	−0.5±0.4	0.1±0.5	-	-	-	-
**DV**	3.1±0.3	2.2±0.3	0.1±0.5	0.4±0.3	-	-	-	-

Table S8: Parameters reflecting nearest neighbor interactions in the indicated peptides. In addition to pPII-β NNIs we list some interaction parameters involving right-handed helical and turn conformations for which the statistical error is not larger than the value itself.

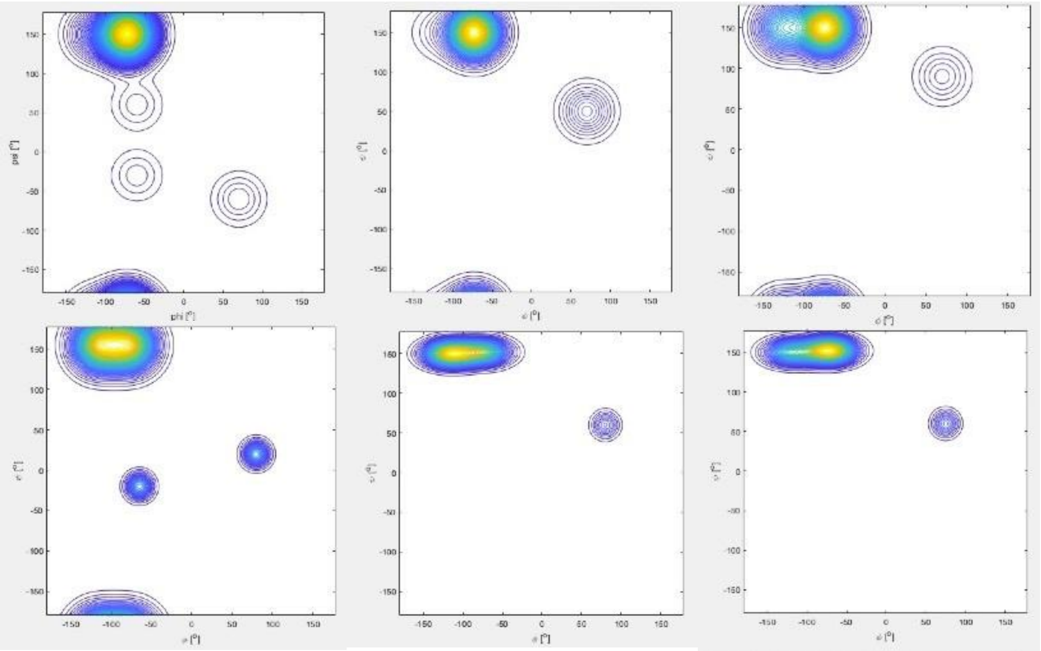

Figure S1: Upper panel: Ramachandran plots of GAG (left), G**A**FG (middle) and GF**A**G (right); Lower panel: Ramachandran plots of GFG (left), GA**F**G(middle) and G**F**AG (left).

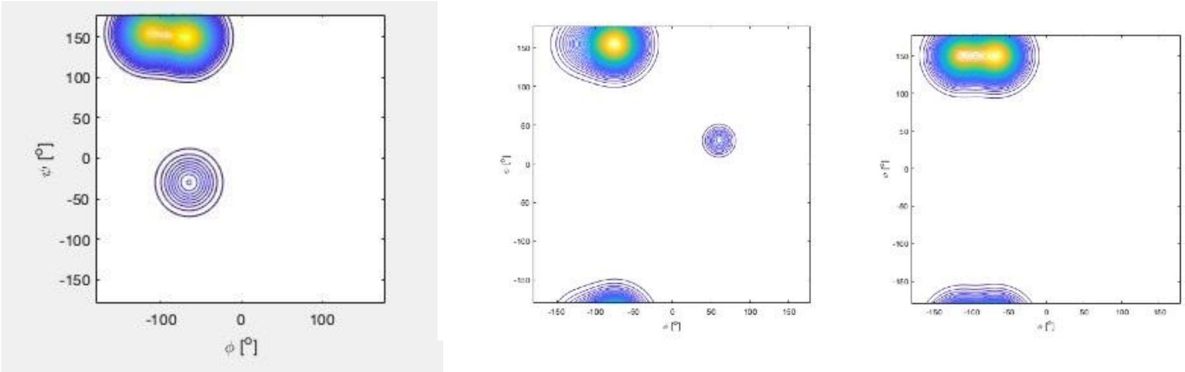

Figure S2: Ramachandran plot of GKG (left), G**K**KG (middle) and GK**K**G (right).
